# Graph Analysis of EEG Functional Connectivity Networks During a Letter-Speech Sound Binding Task in Adult Dyslexics

**DOI:** 10.3389/fpsyg.2021.767839

**Published:** 2021-11-19

**Authors:** Gorka Fraga-González, Dirk J. A. Smit, Melle J. W. Van der Molen, Jurgen Tijms, Cornelis J. Stam, Eco J. C. de Geus, Maurits W. Van der Molen

**Affiliations:** ^1^Department of Psychology, University of Amsterdam, Amsterdam, Netherlands; ^2^Rudolf Berlin Center, Amsterdam, Netherlands; ^3^Department of Child and Adolescent Psychiatry and Psychotherapy, University Hospital of Psychiatry Zurich, University of Zurich, Zurich, Switzerland; ^4^Amsterdam Neuroscience, Amsterdam UMC, Amsterdam, Netherlands; ^5^Neuroscience Campus Amsterdam, VU University, Amsterdam, Netherlands; ^6^Institute of Psychology, Leiden University, Leiden, Netherlands; ^7^Leiden Institute for Brain and Cognition, Leiden University, Leiden, Netherlands; ^8^RID Institute, Amsterdam, Netherlands; ^9^Department of Clinical Neuropsychology and MEG Center, VU University Medical Center, Amsterdam, Netherlands; ^10^Amsterdam Brain and Cognition, University of Amsterdam, Amsterdam, Netherlands

**Keywords:** EEG, networks, dyslexia, letter-speech sound associations, phase lag index, minimum spanning tree (MST)

## Abstract

We performed an EEG graph analysis on data from 31 typical readers (22.27 ± 2.53 y/o) and 24 dyslexics (22.99 ± 2.29 y/o), recorded while they were engaged in an audiovisual task and during resting-state. The task simulates reading acquisition as participants learned new letter-sound mappings via feedback. EEG data was filtered for the delta (0.5–4 Hz), theta (4–8 Hz), alpha (8–13 Hz), and beta (13–30 Hz) bands. We computed the Phase Lag Index (PLI) to provide an estimate of the functional connectivity between all pairs of electrodes per band. Then, networks were constructed using a Minimum Spanning Tree (MST), a unique sub-graph connecting all nodes (electrodes) without loops, aimed at minimizing bias in between groups and conditions comparisons. Both groups showed a comparable accuracy increase during task blocks, indicating that they correctly learned the new associations. The EEG results revealed lower task-specific theta connectivity, and lower theta degree correlation over both rest and task recordings, indicating less network integration in dyslexics compared to typical readers. This pattern suggests a role of theta oscillations in dyslexia and may reflect differences in task engagement between the groups, although robust correlations between MST metrics and performance indices were lacking.

## Introduction

Neuroimaging evidence suggests disrupted functioning in several brain systems involved in reading script in individuals with dyslexia (Shaywitz et al., 2002; Kronschnabel et al., 2014; Žarić et al., 2014) as well as connectivity deficits in brain networks (Pugh et al., 2000; Quaglino et al., 2008; van der Mark et al., 2011; Žarić et al., 2017). Functional neuroimaging studies indicated that dyslexia is associated with disruptions in a broad set of brain systems beyond those typically associated with reading (Finn et al., 2014) and resting-state functional magnetic resonance (fMRI) studies reported that dyslexia is associated with dysfunctional brain connectivity in networks related to reading abilities (Hampson et al., 2006; Koyama et al., 2010, 2013). Another stream of evidence pointed at the potential role of large-scale oscillatory activity networks in dyslexia (e.g., Vourkas et al., 2011; Dimitriadis et al., 2013). In general, oscillations at different frequencies are thought to control communication between anatomical networks (Akam and Kullmann, 2014), enabling different functions under shared anatomical pathways (Fries, 2015). In relation to this, a recent resting-state study using magnetoencephalography (MEG) found support for spatially distinct and behaviorally relevant networks at each classical frequency band (Becker and Hervais-Adelman, 2020).

Previously, we used graph analysis of EEG data to assess the topographical configuration of long-range EEG connectivity at different frequency bands between children (Fraga González et al., 2016) and adults (Fraga González et al., 2018) with dyslexia and typical readers. Graph analysis of the EEG consists of computing a measure of connectivity between each pair of sensors or nodes (*N*) to define an adjacency matrix. The values in this matrix are weights that represent strength of connectivity and they are used to define the network links (*m*). Subsequently, the network can be represented in a graph that allows to calculate metrics describing its topological properties, i.e., how connectivity is organized in the network (e.g., Bullmore and Sporns, 2009; Stam, 2014). These descriptors can be used to characterize the efficiency and specialization of brain systems (both globally and locally) and can help finding new markers of a wide range of disorders (Stam, 2014).

In our resting-state studies on Dutch speakers (Fraga González et al., 2016, 2018), we took advantage of spanning trees (MSTs), a special type of sub-networks which minimizes biases in comparing network metrics between conditions or groups that may differ in overall strength of connectivity (Tewarie et al., 2015). The MSTs contain the highest weights possible without forming any loop or cycle and, in this regard, they can be considered a “connectivity backbone,” which has always the same number of links given a fixed number of nodes (*m* = *N*− 1), assuming that all weight values are unique. Applying this method to resting-state EEG data, our child study revealed statistically significant group differences in the theta (4–8 Hz) band suggesting reduced network integration and less communication between network nodes in children with dyslexia compared to typical readers (Fraga González et al., 2016). A similar study used the same approach on Chinese-speaking children of similar age and found differences between dyslexic and typical readers in the same direction but in MST metrics in the beta band (Xue et al., 2020). They used shorter epoch length and a smaller montage with less electrodes compared to our previous study, which may have contributed to the differences in addition to the different alphabetic systems. Our MST analysis of resting-state EEG data in adults yielded significant network differences between groups in the alpha band (8–13 Hz) and, in contrast to the results observed for children, suggested a more interconnected network configuration in individuals with dyslexia relative to typical readers (Fraga González et al., 2018). These studies yielded no robust associations between graph metrics and cognitive performance. However, a recent study yielded positive results examining the relation between EEG networks and reading skills on L1 Chinese and L2 English-speaking children from first to fifth grade (Lui et al., 2021). The study found that network modularity (derived from the connectivity measure of phase coherence) correlated with Chinese word reading, phonological and morphological awareness, and reading comprehension, but not with any literacy skills in L2 English. That study supported the need to continue exploring the potential of EEG network metrics as predictors of literacy development.

The focus of the current study is a comparison between dyslexic and typically reading adults in EEG data associated with task performance. To date, there are only a couple of studies examining brain networks in dyslexia using a graph theoretic approach to analyze brain activity during task performance. Vourkas and co-workers reported reduced global and local network efficiency in poor readers in the alpha band during a pseudoword reading task and letter-sound naming task (Vourkas et al., 2011). In those tasks participants were asked to read the visually presented pseudowords or to pronounce the sound corresponding to the presented letter, respectively. It should be noted, however, that significant correlations between word reading and graph measures associated with the EEG alpha band were reported only in the more simple letter-sound naming task. In another study, Smith et al. (2018) performed a longitudinal fMRI study examining networks during a rhyming judgment task in young readers over a 2.5 year-span. They reported an association, albeit weak, between a shift in functional segregation (increase in the proportion of functional clusters) and changes in reading skill. A recent study examined fMRI during an auditory rhyming task and a visual spelling task in Chinese children (Mao et al., 2021). The study found differences between poor readers and age-and reading-matched controls in network metrics related to hub properties of frontal and temporal regions relevant for reading, but no relation with behavioral performance was reported. Collectively, the results available to date present little support for a relation between network measures and cognitive skills and/or performance in specific tasks. The current study was designed to investigate just such a relation. More specifically, we examined task-based network organization in dyslexics and typical readers by using an artificial orthography learning task.

The artificial orthography learning task required participants to learn novel letter-speech sound associations by using feedback provided on the screen. The idea behind this task is learning the artificial orthography mimics the initial stages of reading instruction in which correspondences between arbitrary symbols (letters) and speech sounds are established. This specific type of audiovisual integration is considered a key step in fluent reading acquisition by supporting the specialization of visual areas to print, which would ultimately make possible the development of (fluent) sight word reading (Ehri, 2005). Although dyslexic readers seem to be capable of accurately learning letter-speech sound associations, they struggle to automate and sufficiently integrate these associations at the neural level (e.g., Blomert, 2011; Žarić et al., 2014). Our task is inspired by a series of previous studies in which we had children with dyslexia performing a videogame-like task presenting an artificial orthography (Aravena et al., 2013, 2016, 2017). This approach allowed us to obtain an association between task performance and reading skills (Aravena et al., 2017) and responsiveness to reading intervention (Aravena et al., 2016). These findings underline the importance of incidental category (letter-speech sound) learning in developmental dyslexia. Thus, in a another study we developed a feedback learning task in which new symbols are associated with speech sounds (Fraga González et al., 2019). The study found differences on heart-rate changes associated with feedback anticipation, a physiological response previously studied in the context of probabilistic learning (Crone et al., 2004; Kastner et al., 2017). The task design was motivated by the theoretical framework of Holroyd and Coles (2002) for studying error and feedback processing in adapting behavior (Holroyd and Coles, 2002). Their focus was on midbrain dopamine neurons and the of corticostriatal systems in performance adaptation based on prediction error. A set of previous EEG and fMRI studies suggested that dyslexics may process feedback differently compared to typical readers (Horowitz-Kraus and Breznitz, 2011, 2013; Kraus and Horowitz-Kraus, 2014; Horowitz-Kraus and Holland, 2015; Horowitz-Kraus, 2016). The studies, together with some evidence for atypical activations of frontostrital circuits in dyslexia (Krishnan et al., 2016; Hancock et al., 2017b) and reports of potential probabilistic learning impairments (Howard et al., 2006; Gabay et al., 2015; Singh et al., 2018) motivated the examination of this task. The current focus on EEG data associated with the learning of an artificial orthography would provide a window on the alleged dysfunctional neural networks in dyslexia.

To sum up, the main goal of the current study is to compare EEG power, functional connectivity strength and connectivity organization in typical and dyslexic readers during a letter-speech sound binding task. Additionally, we include a resting-state baseline as an additional condition that will allow us to directly compare changes between conditions in the EEG measures, and to test whether group differences are specific to each condition. We then investigate associations between the different EEG measures during task and baseline, and individual differences in task performance and reading skills.

## Materials and Methods

### Participants

Twenty-four dyslexic adults (22.99 ± 2.29 years old) were recruited via a nation-wide center in the Netherlands offering services for individuals with dyslexia. The sample characteristics are summarized in Table 1.^1^ A group of 31 typical readers (22.27 ± 2.53 years old) were recruited via ads at the University and through social networks. Participants with diagnosis of ADHD or other neurological or cognitive impairments were excluded from the sample. Participants were required to have normal or corrected-to-normal vision and Dutch as their primary language. Inclusion criteria for participants with dyslexia were first, persistent reading problems manifested and documented since primary school and with poor response to special support at school for at least 6 months. Second, a diagnosis of dyslexia after assessment at the clinic based on the criteria of DSM-5 (American Psychiatric Association, 2013) and third, a score in a standard word reading fluency test of at least 1 SD below the average of a national normative sample of 16-year-olds. The majority of the participants with dyslexia did not report receiving any specialized treatment for reading disability (a few participants received a 3 months training course for study skills). Ethics approval was obtained from the Ethics Committee of the Faculty of Social and Behavioral Sciences of the University. All participants gave signed consent to their participation in the study.

**TABLE 1:** Sample characteristics and descriptive statistics showing reading scores.

	Typical readers	Dyslexics			
	*M* (*SD*)	*M* (*SD*)	*F*	*p*-value	η^2^
N	31	24			
Sex ratio (m:f)	9:22	12:12			
Handedness (L:R)	1:30	3:21			
Age	22.27 (2.53)	22.99 (2.29)	1.15	0.289	0.02
RAVEN—IQ test^a^	52.52 (4.72)	52.96 (4.71)	0.12	0.732	0.00
1-Min Test –*fluency*^b^	107.32 (8.87)	82.46 (14.14)	63.69	0.000	0.55
Rapid automatized naming^c^					
Letters	16.88 (3.67)	20.88 (4.63)	12.84	0.001	0.19
Numbers	18.45 (4.16)	21.12 (3.95)	5.83	0.019	0.10
Colors	25.42 (4.64)	30.68 (4.58)	17.53	0.000	0.25
Images	28.11 (5.55)	34.81 (6.12)	17.98	0.000	0.25
Total	22.21 (3.27)	26.87 (4.02)	22.45	0.000	0.30

*All raw scores.*

*^a^20 min. time-limited version of RAVEN.*

*^b^Raw score = number of correctly read words within 1 min.*

*^c^Raw score = mean reaction time in sec.*

### Behavioral Measurements

The following tests were taken at the beginning of the session and before attaching the electrodes. Test scores are presented in Table 1. Word reading skills were assessed using a Dutch version of the 1-Min Test (Een-Minuut-Test, EMT; Brus and Voeten, 2010), a time-limited test consisting of a list of 116 unrelated words of increasing difficulty. The number of correctly read words within 1-min serves as reading fluency score (*r* = 0.82, reliability calculated in a normative sample of 16 years old). In addition, participants completed the Rapid Automatized Naming (RAN; van den Bos and Lutje Spelberg, 2010) task that consists of four subtasks: letters, digits, colors, and objects. A sheet containing five items repeated 10 times (arranged in a pseudo-random order) is presented per subtask. Participants are instructed to name the items as quickly as possible, and the time taken to name all items of a sheet provides the subtask’s score (*r* = 0.79–0.86, split-half reliability). Finally, the RAVEN Advanced Progressive Matrices was used to obtain an estimate of fluid IQ (RAVEN APM; Raven and Court, 1998). A 20-min timed version of this test was used as it was previously shown to be a good predictor of the untimed APM (Hamel and Schmittmann, 2006).

### EEG Measurements

#### Recording and Equipment

The EEG recording took place in a dimly lit and sound-proof room. Participants were video-monitored by the lab assistants from an adjacent room to ensure they complied to the instructions and that they did not show behavioral indications of drowsiness or sleep onset during the recording. Participants were seated at approximately 80 cm distance from the computer screen. Their chair was equipped with response buttons at both arms. The EEG session started with preparation and placement of electrodes (lasting around 30 min) and continued with the eyes-open baseline recording and two experimental tasks, which took around 2 h. The order of the experimental tasks was counterbalanced across participants. Following the second experimental task, an additional eyes-open baseline recording was performed to explore reliability and stability of EEG measures within resting state recordings, which falls out of the scope of the current experiment. The current analysis is performed on the data from the initial baseline recording and the main experimental task, i.e., the letter-speech sound binding task (see section “Experimental Task Performance”). The additional experimental task that was part of the recording session, i.e., an audiovisual-binding task, was not used in the present analysis as it is intended for event-related analyses.

The EEG was recorded DC (low-pass: 5th order sync digital filter) with a 2048 Hz sample rate. We used a 64-channel Biosemi ActiveTwo system (Biosemi, Amsterdam, Netherlands). The Biosemi system uses two additional electrodes [Common Mode Sense (CMS) and Driven Right Leg (DRL)] located to the left and right of POz, respectively, which replace the conventional ground electrode. All electrode offsets relative to CMS/DRL were brought within 20 μV in accordance with the manufacturer guidelines. The 64 electrodes were distributed across the scalp according to the extended 10–20 International system (see electrode locations in Supplementary Figure A1) and applied using an elastic electrode cap (Electro-cap International Inc.). Ten external Flat-Type Active electrodes were used. Four were used to record vertical and horizontal electro-oculogram (EOG). They were placed below both eyes aligned with the pupils approximately 3 cm outside both outer canthi of the eyes. Two electrodes were placed at mastoids and two were attached to the earlobes to be used as offline reference signals. Finally, two electrodes were used to record the electrocardiogram (ECG) and were placed at the sternum and between the lower two ribs. The ECG data were not used in the current study. Baseline and experimental task.

During the baseline recording subjects were required to look at the center of the screen during 4 min after making a button-press indicating the start of the period. A gray background was used to minimize glare on the screen and a gray fixation circle with shadowing was placed at the center of the screen to assist participants to fixate their eyes while preventing eye fatigue.

The letter-speech sound binding task is a probabilistic learning task in which subjects learned new visual-sound associations via feedback. We used the current format in a previous study examining differences in overt feedback processing between dyslexics and typical readers (Fraga González et al., 2019). In the trials, participants had to learn whether the letter-like unfamiliar symbol was matched with the simultaneously presented speech sound by pressing Yes or No and receiving feedback after their response. However, feedback was only response-related in half of the trials (consistent trials) while in the other half the feedback was random (inconsistent trials; see below in this section). The visual stimuli consisted of 16 symbols from the Georgian alphabet and the auditory stimuli were 16 Dutch phonemes. The complete list of visual symbols and phonemes used in the task is presented in Supplementary Appendix A. The phonemes were spoken by A native Dutch male speaker. There were three groups of phonemes with different durations; one group of four phonemes had a mean (SD) duration of 172.66 (22.28) ms and another group of four phonemes had a mean (SD) duration of 380.50 (19.47) ms. The third group consisted of eight phonemes with a mean duration of 451.97 (27.69) ms. The visual stimuli were presented using an ASUS VG236H (resolution 1,920 × 1,080) 60 Hz monitor with a Dell Optiplex 760 dual-core 3.0 GHz computer and an ATI HD 6570, 2Gb graphic card. The symbols were presented using ‘‘Arial Unicode MS’’ font (lower case, bold font and font size 60). The software used to present the stimuli was Presentation (Version 18.2^2^). The sound stimuli were presented through padded earphones.

**FIGURE 1:**
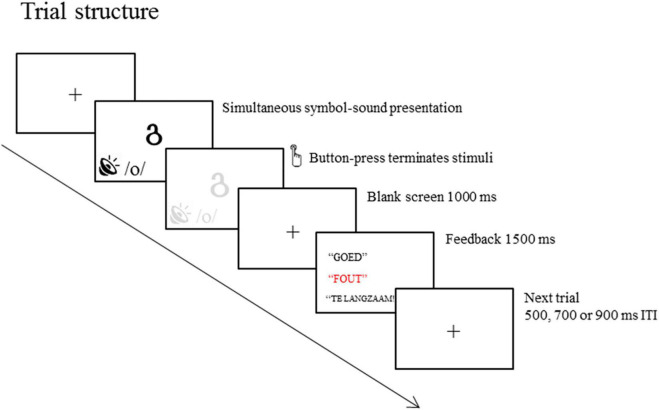
Schematic of a trial in the letter-speech sound binding task. A visual symbol and a phoneme are simultaneously presented and response terminated (only limited by a maximum duration equal to the average reaction time during the practice block + 500 ms). Feedback is presented 1,000 ms after responses to indicate whether the response is correct, incorrect or missed.

A schematic of the trial structure is presented in Figure 1. On each trial, a visual symbol and a phoneme were presented simultaneously. The trials were terminated by the response. The symbols were presented in white on a black background at the center of the computer screen. Participants had to decide whether the symbol and phoneme presented belong with each other by pressing the buttons located at the right and left arms of the chair. The mapping of YES and NO responses to the right and left hand was consistent across blocks for each participant but was counterbalanced across participants. Green and red stickers were placed on the buttons to indicate whether they were YES or NO buttons, respectively. The button-press was followed by blank screen with 1,000 ms duration. The blank screen was followed by feedback “GOED” (correct; presented in white upper case “Times New Roman” font with size 48), “FOUT” (incorrect; presented in red font), or “TE LANGZAAM” (too slow; presented in upper case “Times New Roman” font with size 48). After the feedback screen, a fixation cross was presented during the inter-trial intervals (ITI) with equiprobable durations of 500, 750, or 1,000 ms.

There were 4 blocks of 200 trials. For each block, two visual-sound pairs were consistently matched, and feedback depended upon the response of the participant. The two other visual-sound mappings were inconsistent and followed by random feedback (50% positive and 50% negative feedback). This feedback probability manipulation was included to analyze differential feedback-responses for informative (consistent trials) vs. uninformative (inconsistent trials) responses in a previous study (Fraga González et al., 2019). Note that the current analysis of task performance only uses consistent trials and the EEG analysis is based on a segment during performance that includes both type of trials. Each trial block contained 100 consistent and 100 inconsistent mapping trials presented in random order (50 replications of each individual trial). The duration of a trial block was approximately 14 min. The task began with a practice block of 30 consistent mapping trials. The average reaction time (RT) on correct responses during practice + 500 ms was used to determine the response window. The feedback “too slow” was provided when responses were executed after this window. Participants were told that they should infer the visual-sound associations from the feedback provided to them and that each trial block contained a new set of associations. In addition, they were told that some associations would be more difficult to learn than others.

The whole experimental session took around 3 h and 15 min including the initial behavioral measurements and the montage of electrodes. There were short rests between blocks and between tasks and resting-baselines depending on the needs of the participant. The participants were debriefed at the end of the experiment and received a monetary reward for their services.

#### EEG Preprocessing

**FIGURE 2:**
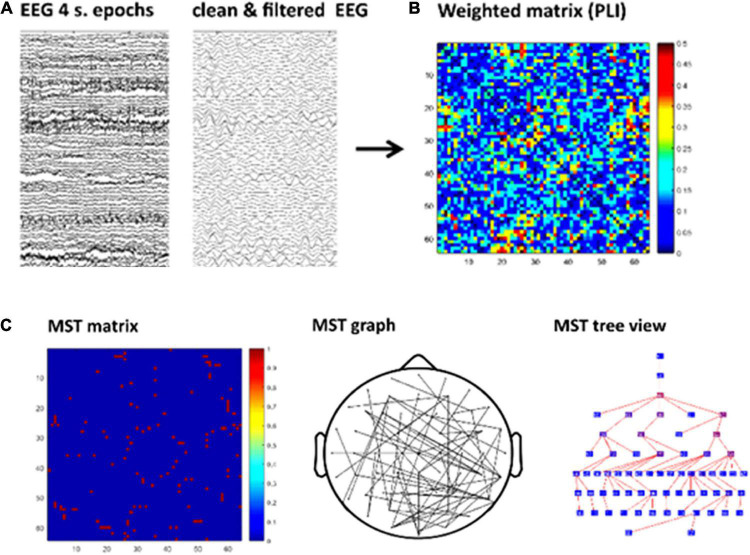
Schematic of the graph analysis. First, visual inspection and Independent Component Analysis (ICA) were applied to remove artifacts. Then data were filtered for each frequency band **(A)**. Second, the functional connectivity matrix based on phase lag index (PLI) is calculated for each frequency band and epoch **(B)**. Kruskal’s algorithm is applied to obtain a minimum spanning tree (MST) matrix (**C**-left) which can be displayed on a scalp projection (**C**-middle). The tree view shows the hierarchical structure of the graph starting from an arbitrary root node. The nodes color map from blue to red represents lower to higher betweenness centrality (**BC**; **C**-right). For illustrative purpose this figure shows the MST obtained from a single epoch in one participant.

The graph analysis followed similar pipeline steps as in our previous study (Fraga González et al., 2016). The sequence of steps of this pipeline are shown in Figure 2. The continuous EEG data were imported in EEGLAB v.12.5.4b, a Matlab-based open toolbox (Delorme and Makeig, 2004). The averaged earlobes were used as off-line reference when importing the data. In the baseline analysis a segment with a duration of 4 min was selected, time-locked to the button press indicating the start of the eyes-open resting-state recording. In the task analysis we took the initial 4 min from the beginning of the task, after the practice period. The data were high-pass filtered at 0.5 Hz using a zero-phase FIR filter and channels containing excessive artifacts were removed from the data to be interpolated later on in the pipeline (see below in this paragraph). The data were then segmented into 60 epochs with a duration of 4 s each. The epochs were visually inspected and those containing artifacts such as head or electrode cable movement and jaw clinching were removed. Subsequently, we performed an Independent Component Analysis (ICA) decomposition (Makeig et al., 1996) in order to remove blinks, eye-movements and other stereotyped artifacts from the data. We used the “runica” algorithm available in EEGlab for ICA decomposition (Lee et al., 1999) and the automatic algorithm ADJUST to identify independent components associated with artifacts (Mognon et al., 2011). The algorithm uses artifact-specific spatial and temporal features to detect artifactual components and has been previously validated (Mognon et al., 2011). After removing the independent components selected by the algorithm, data for typical readers and dyslexics were reconstructed based on a mean (SD) of 52.67 (7.82) and 49.37 (14.02) components in the task and 52.23 (4.58) and 51.29 (5.90) components in the baseline, respectively. Afterward, the data from previously removed channels were interpolated using a spherical spline interpolation method (Perrin et al., 1989). Finally, for each condition (baseline and task) a total of 30 epochs, each with a duration of 4 s, were selected per participant,^3^ down-sampled to 1024 Hz and exported to ASCII files for the subsequent EEG analyses.

The ASCII files were imported in Brainwave v0.9.152.4.1 (developed by C.S.; freely available at http://home.kpn.nl/stam7883/brainwave.html) where data were re-referenced to the average of all scalp channels and filtered for each frequency band (see section “Spectral Power”) before performing subsequent analyses.

#### Spectral Power

We calculated spectral power in each epoch using Fast Fourier Transformation (FFT) with a frequency resolution of 1 / 4 s = 0.25 Hz. The power spectra were averaged across segments and all the groups of electrodes described in section ‘‘EEG Preprocessing.’’ The ‘‘area under the curve’’ values were calculated for the following frequency bands: delta (0.5--4 Hz), theta (4--8 Hz), alpha (8--13 Hz),^4^ and beta (13–30 Hz). Relative power was computed as the ratio of the power of the corresponding band and the total power.

#### Functional Connectivity

We used the Phase Lag Index (PLI) to calculate functional connectivity between all pairs of electrodes for each frequency band and epoch. In contrasts to other connectivity measures like phase coherence, the PLI reduces the effect of volume conduction by ignoring zero and π phase differences (Stam et al., 2007). It captures the asymmetry of the distribution of instantaneous phase differences, which are determined using the Hilbert transformation (Stam et al., 2007). A symmetric distribution centered around zero may indicate spurious connectivity and a flat distribution indicates a lack of connectivity. A deviancy from a symmetric distribution indicates dependency between sources. The PLI is obtained from time series of phase differences Δϕ (*t*_k_), k = 1…*N* by means of:

PLI=|<sign[sin(Δϕ(t)k)]>|

Here “sign” is the signum function. The PLI ranges between 0 and 1. A value of 0 means no coupling or coupling with a phase difference centered around 0 (mod π). A value of 1 indicates perfect phase locking at a value of Δϕ different from 0 (mod π). Thus, PLI values closer to 1 indicate stronger nonzero phase locking. In the current analysis we use the term mean total PLI when referring to the average of the PLI between all pairs of electrodes.

#### Minimum Spanning Tree Analysis

For our network analysis, we calculated a Minimum Spanning Tree (MST) for each connectivity matrix (see Figure 2). We used the MST as it allows for direct group or condition comparisons minimizing the bias caused by differences in connectivity strength (e.g., Stam et al., 2014). The MST is a unique sub-graph based on a weighted matrix that connects all nodes of the network without circles or loops. Importantly, the MST always contains *m* = *N*−1 links, where *N* is the number of nodes. The MST was constructed by applying Kruskal’s algorithm (Kruskal, 1956) which iteratively selects the links with the lowest distance (i.e., lowest weights) and adds the link to the tree only if no loops are created. The result is a graph without cycles or loops in which all nodes are connected. In our MST computation, we define a link weight as 1-PLI. Thus, the MST represents the sub-network with maximum connectivity.

There are a various MST metrics that are used to describe the topological properties of the tree (Stam et al., 2014). We examined the following metrics: degree, leaf fraction, diameter, eccentricity, betweenness centrality (*BC*), tree hierarchy (*Th*), degree correlation (*R*), kappa and mean. The degree of a node refers to its number of links, and the leaf fraction represents the number of nodes (*N*) on the tree with degree = 1. The leaf number has a lower bound of 2 and an upper bound of *N*− 1. It presents an upper bound to the diameter of the MST, which is the largest distance between any two nodes of the tree. The upper limit of the diameter is *d* = *m*− *L* + 2, where *m* refers to the number of links on the tree. This formula implies that the largest possible diameter will decrease with the increasing leaf number. Eccentricity of a node is defined as the longest distance between that node and any other node and is low if this node is central in the tree. The *BC* of a given node *u* is the number of shortest paths between any pair of nodes *i* and *j* that are running through *u*, divided by the total number of paths between *i* and *j*. The *BC* value ranges between 0 and 1 and relates to the importance of a node within the network. The nodes with the highest *BC* have the highest load, i.e., the highest number of shortest paths between any two nodes run through these high *BC* nodes. For example, a central node with a *BC* of 1 could be easily overloaded. Degree, eccentricity and *BC* are different measures for relative nodal importance and may indicate the critical nodes in a tree. The measure of tree hierarchy *T*_*h*_ reflects a balance between efficient communication and prevention of overload of hub nodes, reflected, respectively, by small diameter and a maximal *BC*. This balance is proposed to be important for optimal network performance (Boersma et al., 2013) and is defined as:

$$T_{H}=\frac{L}{2\,m\,B\,C_{m\,a\,x}}$$

Where *L* is leaf fraction and *m* the number of links. Further, the degree correlation *R* is an index of whether the degree of a node is correlated with the degree of its neighboring edges to which it is connected. The *R* is quantified by computing the Pearson correlation coefficient of the degrees of pairs of connected nodes (Newman, 2003). If *R* > 0 the graph is considered assortative, and if *R <* 0 disassortative. Kappa is the width of the degree distribution and relates to spread of information across the tree (Stam et al., 2014). High kappa indicates the presence of high-degree nodes, which facilitate synchronization of the tree but also increase the network’s vulnerability if a hub is damaged (Otte et al., 2015). Finally, we computed the MST mean, that is the mean of the PLI weights of the tree.

### Statistical Analysis

Experimental task performance was evaluated by calculating accuracy and speed on consistent-mapping trials across four bins of 25 trials for each trial block. These data were also averaged across 4 experimental blocks. Mixed-model ANOVAs were used to compare groups in accuracy and reaction times across blocks with the within-subjects factor *bin* (1–4). As behavioral, performance summary measures to correlate with EEG measures we computed the total accuracy average as well as the average RT of correct responses. A more detailed analysis of performance in this task, together with an additional control audiovisual binding task can be seen in Fraga González et al. (2019).

Our main EEG analysis consisted of a mixed ANOVA comparing the groups in task data. Additionally, we performed the same comparisons in the resting-state baseline data. A third analysis explored interactions between group and difference in task vs. resting state with mixed ANOVAs with the within-subjects factor *condition* (2 levels; baseline and task) and the between-subjects factor *dyslexia*. Moreover, regression analysis was performed between PLI and relative power. Greenhouse-Geisser correction of degrees of freedom was used to calculate *p*-values when the assumption of sphericity was violated (Greenhouse and Geisser, 1959). To account for the multiple comparisons performed in network metrics we used False Discovery Rate (FDR; Benjamini and Hochberg, 1995). Given the correlation between network metrics we accepted a 10% of false discoveries (*q* = 0.10), we also report a more stringent FDR correction at *q* = 0.05 (see footnotes in the corresponding tables).

Finally, we used stepwise multiple linear regression in the two groups separately to explore whether EEG power, connectivity and graph metrics could predict task performance, cognitive skills and age. The inclusion criteria for the EEG variables to be included in the regression models were *p* < 0.05 and the exclusion criteria was *p* > 0.10.

## Results

### Cognitive Measures

The scores for reading accuracy and speed measures are shown in Table 1. The dyslexic group performed significantly worse than typical readers on both reading tests and the deficit was more pronounced on the word identification task. The two groups were comparable in non-verbal IQ and age.

### Experimental Task Performance

The descriptive statistics of the performance data (accuracy and RTs averaged across blocks) are presented in Table 2 and Supplementary Figure A2 and Supplementary Table A2 shows the extended descriptives per block for the consistent trials). The ANOVA performed on accuracy revealed a significant main effect of Bin, *F*(3, 159) = 106.89, *p* < 0.001, η^2^ = 0.67, indicating that accuracy increased with time-on-task, illustrating probability learning. There were no significant group differences or interactions with the factor dyslexia, *p*s > 0.124. The follow-up pairwise comparisons between bins across groups showed significantly increased accuracy from bin 1 to bin 2 (mean difference 10.53, *p* < 0.001), but not between bin 2 and 3 or bin 3 and 4 (*p*s > 367). The mean accuracy per bin and group are presented in Table 2 and the Supplementary Figure A2. The ANOVA performed on RTs yielded a trend for slower responses in dyslexics relative to typical readers across all four bins, *F*(1, 53) = 3.85, *p* = 0.055, η^2^ = 0.07, all other *p*s > 0.121. The RTs are shown in Table 2 (see also Supplementary Figure A2).

**TABLE 2:** Task performance in letter-speech sound task for the consistent trials.

		Typical readers (*N* = 31)		Dyslexics (*N* = 24)	
		Accuracy	Reaction time	Accuracy	Reaction time
		*M* (*SD*)	*M* (*SD*)	*M* (*SD*)	*M* (*SD*)
Letter-speech sound binding task					
*Mean over 4 blocks*	Bin1	76.81 (9.90)	886.63 (124.73)	74.71 (9.53)	928.32 (131.80)
	Bin2	86.71 (8.65)	861.42 (138.27)	85.88 (10.27)	934.45 (134.80)
	Bin3	87.68 (9.80)	868.31 (144.24)	87.58 (8.27)	932.66 (118.45)
	Bin4	87.39 (9.92)	861.05 (131.42)	89.08 (9.22)	945.84 (112.73)

*Bin 1 = trials 1–25; Bin2 = trials 26–50; Bin3 = trials 51–75; Bin4 = trials 76–100. Reaction times to correct responses in milliseconds.*

*Accuracy = percentage of correct responses.*

### Group Differences in EEG

We performed a mixed ANOVA with the factor condition (task, baseline) to examine group differences during task and resting state, and the interaction between these factors.

#### Relative Power

**FIGURE 3:**
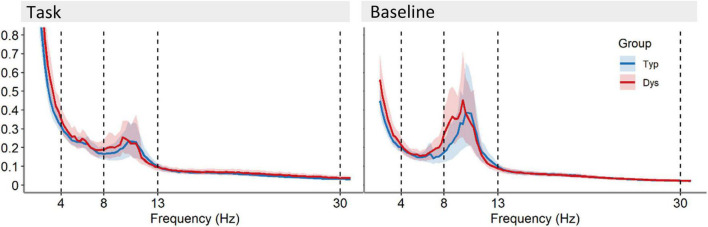
Power spectra averaged across 64 EEG scalp channels with 95% CI for the recording during task and the baseline recording for dyslexics (red) and typical readers (blue). Vertical dotted lines indicate the boundaries for the frequency bands at 4, 8, 13, and 30 Hz.

**FIGURE 4:**
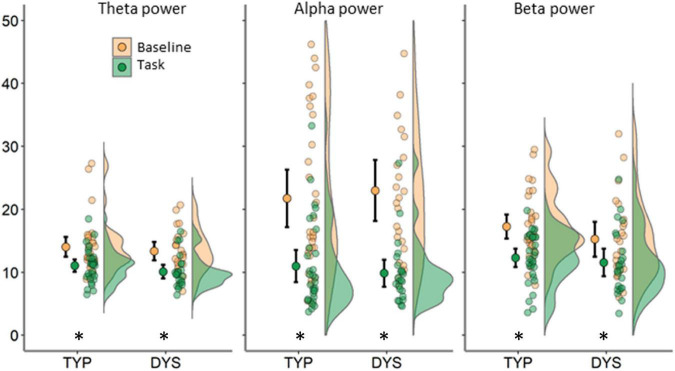
Relative power averaged across 64 scalp electrodes for each condition (indicated by color) and frequency band. Each plot shows data for typical readers at the left side and for dyslexics at the right side. Error bars represent 95% CI. Asterisks indicate significant differences between conditions at *p* < 0.01.

The FFT power spectra per condition and group are presented in Figure 3. As expected, there were significant differences between the task and baseline recordings in theta [*F*(1, 53) = 41.83, *p <* 0.001, η^2^ = 0.44], alpha [*F*(1, 53) = 109.88, *p <* 0.001, η^2^ = 0.68] and beta relative power, *F*(1, 53) = 32.10, *p <* 0.001, η^2^ = 0.38. Relative power was significantly larger in the baseline compared to the task (see Figure 4). There was no evidence for significant interactions or main effect of group in these analyses, *p*s < 0.258.

#### Phase Lag Index Connectivity

**FIGURE 5:**
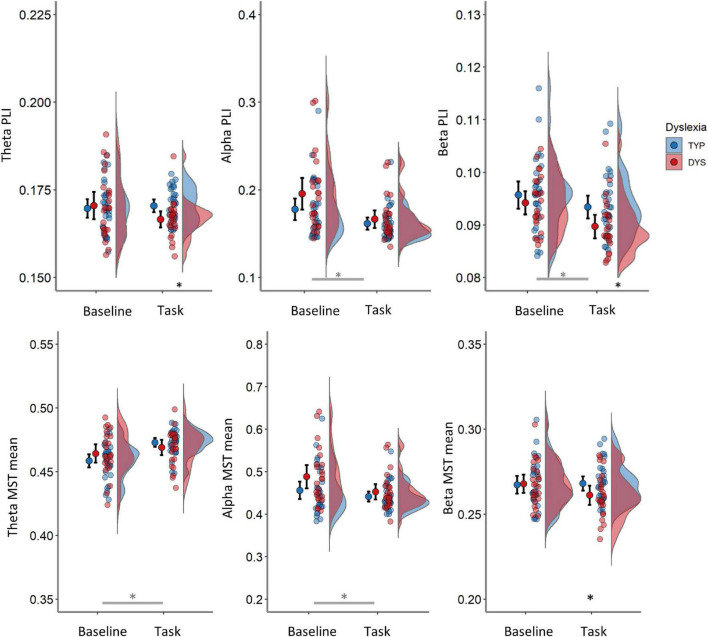
Averaged PLI (top row) and mean MST (bottom row) in the theta (left panels), alpha (middle panels) and beta band (right panels) per condition. Red color indicates data from dyslexics and blue those from typical readers. Error bars represent 95% CI. Black asterisks indicate comparisons between groups significant at *p* < 0.05. Gray asterisk indicates main effect of condition at *p* < 0.05. TYP, typical readers; DYS, dyslexics.

The main analysis on PLI is presented on Table 3 (see Supplementary Table A3 for all tests that were performed). There was a significant main effect of condition in the alpha band indicating larger PLI in the baseline compared to task [*F*(1, 53) = 29.02, *p <* 0.001, η^2^ = 0.35], but no interactions or main effect of group in that band, *p*s > 0.119. A significant effect in the same direction was detected in the beta band [*F*(1, 53) = 24.64, *p <* 0.001, η^2^ = 0.32], together with a trend for lower values over both conditions in dyslexics compared to typical readers, *F*(1, 53) = 3.1, *p* = 0.084, η^2^ = 0.06. In the theta band there was no main effect of group or condition (*p*s > 0.151) but, there was a significant interaction between condition and group [*F*(1, 53) = 4.45, *p* = 0.040, η^2^ = 0.08], indicating lower PLI in dyslexics vs. typical readers during the task but not in the baseline. The task vs. baseline in dyslexics but not in typical readers (see Figure 5 and Table 3).

**TABLE 3:** Group (dyslexics, typical readers) and condition (baseline, task) comparisons for PLI and MST metrics.

		Within-subjects						Between-subjects	
			Condition			Condition × Dyslexia		Group	
		ΔTask	*F*	*p*	η^2^	*F*	*p*	*F*	*p*
*Theta*	PLI		2.12	0.151	0.04	**4.45**	**0.040**	1.04	0.313
	Degree	↓	**18.06**	0.000**	**0.26**	*3.45*	*0.069*	1.60	0.211
	Leaf	↓	**59.98**	0.000**	**0.53**	*3.60*	*0.064*	1.10	0.163
	*T*_H_	↓	**49.70**	0.000***	**0.48**	**2.09**	**0.038**	2.47	0.122
	*R*	↓	**42.86**	0.000**	**0.44**	*3.20*	*0.080*	**6.36**	**0.015**
	MST mean	↑	**22.29**	0.000**	**0.30**	**5.70**	**0.021**	0.90	0.766
*Alpha*	PLI	↑	**29.02**	0.000**	**0.35**	2.25	0.140	2.50	0.119
	Degree	↓	**74.02**	0.000**	**0.58**	**4.09**	**0.048**	1.29	0.261
	Kappa	↓	**98.80**	0.000**	**0.65**	**5.68**	**0.021**	1.47	0.230
	MST mean^a^	↓	**15.21**	0.000**	**0.22**	2.63	0.111	*3.30*	*0.075*
*Beta*	PLI	↓	**24.64**	0.000**	**0.32**	2.66	0.109	*3.10*	*0.084*
	MST mean	.	*3.42*	*0.070*	*0.06*	**5.27**	**0.026**	1.04	0.312

PLI, phase lag index; Ecc, Eccentricity; BC, betweenness centrality; T_H_, tree hierarchy; R, degree correlation; ↑ indicates increase in task vs. baselines; ↓ indicates decrease in task vs. baselines. ^a^Direction of effect differs between frequency bands. **Significant effects after FDR correction at q = 0.05; bold text represents significant effects at uncorrected p < 0.05; italic text represents trends.

The condition and group interactions were followed by group comparisons in task and baseline data separately (see Table 4 and Supplementary Table A4). In the task, PLI theta was significantly lower in dyslexics compared to typical readers, *F*(1, 53) = 7.63 *p* = 0.008, η^2^ = 0.13 (see left panel in Figure 5). The mean (SD) total PLI theta was 0.167 (0.005) and 0.170 (0.005) for dyslexics and typical readers, respectively. The mean total PLI beta was lower in dyslexics compared to typical readers, *F*(1, 53) = 5.88, *p* = 0.019, η^2^ = 0.10. The mean (SD) total PLI beta was 0.090 (0.005) and 0.093 (0.006) for dyslexics and typical readers, respectively. The analysis of the baseline data showed no evidence for significant group differences in PLI, although there was trend for stronger alpha connectivity in dyslexics vs. typical readers at *p* = 0.091, all other *p*s > 0.388.

**TABLE 4:** Group comparisons per condition for PLI and network metrics.

		*Task*			*Baseline*		
		*F*	*p*	*Dys* vs. *Typ*	*F*	*p*	*Dys* vs. *Typ*
*Theta*	PLI	**7.63**	**0.008**	<	0.14	0.715	
	*T*_H_	*3.91*	*0.053*		0.37	0.544	
	*R*	**7.33**	0.009*	<	1.23	0.272	
*Alpha*	PLI	0.78	0.383		*2.97*	*0.091*	
	Degree	0.77	0.383		*3.10*	*0.084*	
	Kappa	0.00	0.948		*3.71*	*0.060*	
*Beta*	PLI	**5.89**	**0.019**	<	0.76	0.388	
	MST mean	**4.27**	**0.044**	<	0.23	0.879	

*PLI, phase lag index; Dys, dyslexics; Typ, typical readers; T_H_, tree hierarchy; R, degree correlation.*

*Significant effects after FDR correction at q = 0.10; italic text represents trends; bold text represents significant effects at uncorrected p < 0.05.

#### Minimum Spanning Tree Network Metrics

The results of the main ANOVA on MST metrics revealed significant group differences across conditions (see Table 3 and Supplementary Table A3). Dyslexics showed lower theta degree correlation, i.e., lower network integration, over both task and baseline recordings, *F*(1, 54) = 6.36, *p* < 0.015. In addition, there were significant main effects of condition for all MST metrics except for betweenness centrality in theta and MST mean in beta. The largest effect sizes for the change across conditions were found on degree (alpha) leaf fraction (theta, alpha and beta), kappa (alpha), tree hierarchy (theta and alpha) and degree correlation (theta and alpha) with partial eta-squared > 40. The direction of these differences suggests a less integrated network configuration in task compared to the pre-task baseline. There were significant interactions between condition and dyslexia for theta tree hierarchy, alpha kappa and beta MST mean and theta MST mean (see Table 3). The follow-up analyses on these interactions are presented in Table 4 (and Supplementary Table A4). These analyses showed a trend for lower the tree hierarchy in dyslexics compared to typical readers during task [*F*(1, 53) = 3.92, *p* = 0.053] but not in the baseline *p* = 0.544. For alpha kappa, dyslexics showed a trend for larger kappa than typical readers in baseline, *p* = 0.060, that was absent in the task 0.948 (see Table 4).

### Relation Between EEG Measures and Cognitive Performance

Stepwise regressions examined whether EEG power, connectivity and graph metrics could predict task performance, cognitive skills and age in the two groups (*p* < 0.05 for inclusion of EEG variable in the model, *p* > 0.10 for exclusion). The results are presented in Table 5.

**TABLE 5:** Significant stepwise regressions of performance, age and cognitive skills to EEG metrics.

			SE	Adj. *R*^2^	*ΔR* ^2^	*F change*
**Typical readers**						
Mean RT	*Model 1*	Task theta power	118.47	0.139	0.168	5.84*
Mean accuracy	*Model 1*	Baseline Beta BC	8.31	0.124	0.153	5.23*
	*Model 2*	+ Baseline Alpha BC	7.62	0.264	0.160	6.51*
	*Model 3*	+ Task Alpha BC	6.90	0.396	0.144	7.15*
	*Model 4*	+ Task beta Th	5.64	0.596	0.194	14.42***
RAN numbers	*Model 1*	Task theta Mean	3.93	0.105	0.135	4.51*
RAN colors	*Model 1*	Baseline Theta PLI	4.22	0.172	0.199	7.21*
	*Model 2*	+ Baseline Beta BC	3.92	0.288	0.136	5.73*
RAN images	*Model 1*	Task alpha Th	5.14	0.143	0.171	5.99*
	*Model 2*	+ Task beta Kappa	4.84	0.240	0.120	4.73*
	*Model 3*	+ Baseline Beta BC	4.58	0.320	0.097	4.30*
	*Model 4*	+ Baseline Theta PLI	4.30	0.402	0.094	4.71*
	*Model 5*	+ Baseline Alpha R	3.97	0.488	0.092	5.37*
	*Model 6*	+ Task theta R	3.61	0.577	0.088	6.25*
**Dyslexics**						
Age	*Model 1*	Task alpha degree	1.73	0.432	0.456	18.48***
IQ	*Model 1*	Baseline Alpha Th	4.08	2.51	0.284	8.72**
RAN total	*Model 1*	Task theta BC	3.45	0.261	0.293	9.13**
	*Model 2*	+ Baseline Theta Diameter	3.11	0.401	0.160	6.16*
	*Model 3*	+ Baseline Theta PLI	2.80	0.513	0.123	5.83*
	*Model 4*	+ Task alpha power	2.49	0.584	0.080	4.40*
RAN numbers	*Model 1*	Task theta BC	3.47	0.226	0.260	7.72*
	*Model 2*	+ Baseline Beta R	3.01	0.469	0.210	8.30**
	*Model 3*	+ Task Beta Leaf	2.75	0.579	0.110	5.21*
	*Model 4*	+ Task Alpha R	2.53	0.661	0.081	4.56*
RAN colors	*Model 1*	Baseline Beta BC	4.13	0.187	0.223	6.30*

SE, standard error of the estimate; Adj. R^2^, adjusted R squared; ΔR^2,^ change in R squared; + indicates variable is added to those of preceding models. BC, betweenness centrality; PLI, phase lag index; Th, tree hierarchy; R = degree correlation. *p < 0.05; **p < 0.01; ***p < 0.001.

In typical readers there were significant regression models including different combinations of EEG measures for task RT and accuracy (maximum adjusted *R*^2^ = 0.596 in model 4 for mean accuracy) and the RAN subtasks of numbers, colors and images (maximum adjusted *R*^2^ = 0.577 in model 6 for RAN images). In the dyslexic group, age was predicted by alpha degree during task (adjusted *R*^2^ = 0.432 in model 1), IQ by baseline alpha tree hierarchy (adjusted *R*^2^ = 0.251 in model 1) and RAN total, numbers and colors were predicted by several EEG variables combined (maximum adjusted *R*^2^ = 0.661 in model 4 for RAN numbers). To sum up, we did not a find a consistent pattern of associations between a specific set of EEG measures and individual performance and cognitive characteristics. Multiple combinations of EEG power, connectivity and graph metrics from all three frequency bands contributed to predict several individual characteristics, which differed between the groups. A similar result was found when using the data of both groups in the analysis, this is presented in Supplementary Table A5.

### Association Between Phase Lag Index and Spectral Power

**FIGURE 6:**
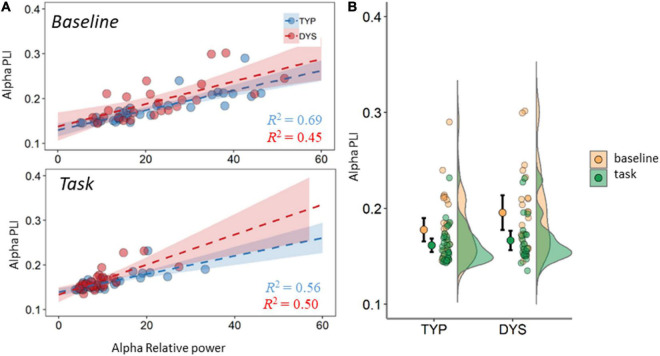
Averaged PLI in the alpha band **(A)** mean PLI plotted against mean relative power. Dashed lines are regression lines for typical readers (blue) and dyslexics (red). **(B)** Mean PLI for task and baseline recordings. DYS, dyslexics; TYP, typical readers. Error bars indicate 95% CI.

Since there were significant differences in power, connectivity and MST measures between baseline and task, we also examined the relation between PLI and relative power for each band and condition. The regression analysis for the theta band revealed a significant relation between PLI and relative power for baseline theta (*R* = 0.55, *R*^2^ = 0.31, *p* < 0.001) and task theta (*R* = 0.39, *R*^2^ = 0.15, *p* = 0.003). The same pattern was observed for the alpha band; baseline (*R* = 0.73, *R*^2^ = 0.54, *p* < 0.001) and task (*R* = 0.86, *R*^2^ = 0.47, *p* < 0.001). It can be observed that the strength of the correlation between PLI and power differed between the groups. In theta band, the strength of this relation was moderate in dyslexics and in typical readers the relation was weak or negligible (these results are plotted in the Supplementary Figure A3). In the alpha band, typical readers show moderate to strong correlations between PLI and power, while in dyslexics these values were lower. This result is plotted in Figure 6, which also shows the regression lines and coefficients per group. There was no significant relation between PLI and relative power for the beta band.

## Discussion

The aim of the current study was to examine whether letter-speech sound binding task-based EEG network measures could discriminate dyslexics from typical readers and/or relate to reading abilities or task performance. In addition, task vs. resting-state differences in functional connectivity and graph measures were explored. The latter examination allows us to extend our discussion on reliability and dependency on FFT power issues that can affect interpretation.

### Group Differences in Theta and Beta Connectivity During Task

We found task-specific group differences in theta connectivity. Dyslexics showed lower mean connectivity in theta compared to typical readers. In general terms, oscillatory activity in lower frequency bands such as theta is proposed to reflect long distance synchronization while in higher frequencies it would relate to shorter distances or smaller networks (Buzsáki and Draguhn, 2004). Here, due to our focus on large scale networks, we used the PLI measure which is shown to be more robust against group differences in volume conduction than other measures, albeit at the expense of a higher risk of missing meaningful phase differences at short distances (Stam et al., 2007). Our results regarding PLI theta suggest decreased overall long-range connectivity in dyslexics during the current task simulating reading acquisition. The available literature on functional connectivity has revealed mixed alterations in dyslexia, showing evidence for both increased and decreased connectivity depending on region and task (Marosi et al., 1995; Nagarajan et al., 1999; Shiota et al., 2000; Arns et al., 2007; Dhar et al., 2010). Our finding would be in agreement with previous findings of impaired functional connectivity in dyslexics compared to typical readers across major pathways (e.g., Finn et al., 2014) and the hypothesis that general oscillatory mechanisms may play a role in dyslexia (Hancock et al., 2017a).

Another result in the theta band that emerged from the task data refers to the lower degree correlation in dyslexics compared to typical readers. The graph metric of degree correlation reflects the extent to which connected nodes have similar degrees. A previous EEG study found lower degree correlation (*R*) in alpha between patients with Alzheimer and controls (de Haan et al., 2009). That finding was interpreted as indicating loss of network structure in the patient group. In addition, an MEG study found an association between lower *R* and decreased neurocognitive performance in glioma patients (Bosma et al., 2009). In that study higher *R* in delta was associated with better attentional functioning and *R* in lower alpha was associated with verbal memory performance. The *R* of a randomly organized network is close to 0, thus the authors interpreted that result as reflecting deviation from optimal organization of a network. The current group differences in *R* theta might therefore indicate a suboptimal network structure in dyslexics during task performance. However, we did not find a reliable association between *R* and performance measures in the current task. Moreover, the groups did not show significant differences in task performance, although the current trend for longer RTs in dyslexic readers reached statistical significance in our previous work using this task in a sample largely overlapping the present (Fraga González et al., 2019). It is possible that our performance analysis did not capture differences in specific components of learning that impose different attentional and cognitive demands in dyslexics and typical readers and can thus be related to theta networks (this is further discussed in the section “Limitations”). Theta oscillatory activity has been previously associated with working memory and attentional functioning (Klimesch, 1999; von Stein and Sarnthein, 2000; Gootjes et al., 2006). More relevant to the present results, theta activity has been linked to dyslexia and reading difficulties in other studies (Arns et al., 2007; Spironelli et al., 2008; Goswami, 2011; Fraga González et al., 2016). In our previous work using resting-state data showed that several MST metrics in theta related to network integration could discriminate between typical readers and dyslexics in children (Fraga González et al., 2016) but not in adults (Fraga González et al., 2018). The current findings expand previous results and support the involvement of theta oscillations in cognitive performance and dyslexia.

Further, the analysis in the beta band revealed group differences in PLI, suggesting that in dyslexics connectivity was lower during task compared to typical readers. Although the role of beta band activity is less clear, Engel et al. (2001) suggested that beta activity might be associated to maintenance of motor actions and cognition. Specifically, that report indicated a role of beta synchronization in top-down prediction. It is thus possible that our finding in the beta band relates to differences in task engagement between the groups, although we did not find correlational evidence to further support this interpretation. The following discussion on task vs. resting-state comparisons and limitations to our analytic approach to task recording is also relevant to this interpretation.

### Network Configuration Differences in Task and Resting-State

The comparisons across conditions revealed a less integrated network configuration and reduced mean connectivity during task performance compared to baseline in all frequency bands and for both groups. This overall pattern may reflect more specialized processing, i.e., recruitment of specific networks, which would be expected during performance of a specific task. In a previous study, surface EEG signals were compared between rest and during a mental arithmetic task in adults vs. children using both static and time-varying networks (Dimitriadis et al., 2015). In that study, inconsistent with our findings, the static network measures of local and global efficiency did not show sensitivity in the task vs. resting-state comparisons, although such difference was found in dynamic measures related to transitivity between network “microstates.” A potential reason for the apparent discrepancy in the results is the network construction approach (weighted graph derived directly from the connectivity measures vs. MST graph in the current study). Another issue complicating a direct comparison refers to the task nature (arithmetic vs. association learning) and difficulty: ceiling levels of performance are reported in their study while our behavioral analysis suggests that our task was, to some extent, more challenging to participants. Additional aspects of task design, like trial and feedback structure might have contributed also to these differences. Interestingly, the impact of task difficulty in several MST metrics has been previously studied in another experiment using an arithmetic task (Vourkas et al., 2014). That study suggested more distributed networks in theta and more integrated configuration in alpha with increasing task difficulty, as well as significant, albeit weak, correlations between graph measures and task performance. Unfortunately, our current design did not include a difficulty manipulation. We did find statistically significant association between theta power during task and performance RT in typical readers that would point at the same direction in that group. However, the low strength of this association does not warrant further interpretation.

Another relevant issue when interpreting task vs. baseline network changes relates to FFT power. Our regression analysis (see Figure 6) shows that there is a moderate influence of power in the estimation of functional connectivity. This seems especially relevant in alpha where a large drop in power is expected during task- vs. resting-state. This is evident in the mean FFT plots in Figure 3 as well as in the density plots in Figure 4, showing large individual variability in relative alpha power for baselines compared to a narrower distribution with lower values for task data. This result is in agreement with the proposed inhibitory role of alpha activity (Jensen and Mazaheri, 2010; Mathewson et al., 2011). Despite this association and in support of the additional value of mean connectivity measures, there were no significant group differences were found in spectral power. However, such group differences in alpha power were reported during a visuospatial orientation task (Van der Lubbe et al., 2019) and in numerous resting-state studies, although with inconsistent findings (see summary table in Lui et al., 2021). An important consideration derived from the above studies and our regression analysis is the necessity for examining spectral power, often underreported in network studies. This was further brought into attention in a short communication (Demuru et al., 2019).

### Limitations

There are some limitations to note for the present this study. A first limitation relates to EEG montage and sensor-level analysis. But as mentioned in our previous work, our choice of PLI as connectivity measure aims at minimizing the impact of volume conduction and it seems to allow reliable network topology estimates (Lai et al., 2018). A second, more specific limitation, is the analysis of task-related activity using measures which have been primarily applied to resting-state data. Here we used a rather “coarse” approach, analyzing epochs derived from a broad segment of the task recording matched in duration to our 4 min baseline period. This approach, used in previous work (Vourkas et al., 2014) ignores the structure of events or task trials and assumes that in order to perform the task, participants must sustain a cognitive and attentional state that is relatively constant during the block. However, it is obvious that levels of concentration, alertness, processing speed and fatigue among other factors, may vary at different levels from each block to the whole experimental session. Other studies used a more event-related approach segmenting a time window preceding and following the event (Vourkas et al., 2011; Wang et al., 2016) which has another set of problems, i.e., related to the amount of data points per segment and network stability (Fraschini et al., 2016). Direct comparisons between these two methods would require a more constrained design beyond the scope of the present work. Finally, using more advanced models for analyzing task performance may yield behavioral indices of the trial-by-trial learning process that can be better associated with large-scale oscillatory activity. The contribution of model-based cognitive neuroscience in the context of networks and dyslexia remains underexplored.

## Conclusion

We found reduced theta connectivity strength during task in dyslexics compared to typical readers and trends for group differences in both task and resting state in several network metrics. These differences were not detected when examining EEG power and support that overall connectivity in theta activity during task performance may be implicated in dyslexia. This is also suggested by the differences between task and resting-state in theta connectivity that also seem to diverge between the groups. However, it remains unclear whether these group differences in EEG connectivity reflect atypical activations of specific hub regions, recruitment of different networks, or they involve more widespread oscillatory mechanisms. More spatially resolved techniques might clarify some of these questions. In addition, the EEG group differences were not reflected in learning differences during the task and a robust association between functional network metrics and cognitive performance remained elusive. Model-based analyses and tasks that can capture variability in reading skills will be important to further develop a cognitive interpretation of these EEG measures. In this direction, other network metrics that can be derived across frequencies and tasks may offer more promising neural correlates of literacy (Lui et al., 2021). Further, our findings emphasize the need to consider the unique contribution of each set of measures (i.e., overall strength of functional connectivity and graph-derived metrics), their intercorrelation across recordings, as well as the influence of spectral power. This would benefit the interpretability of network findings in future studies.

## Data Availability Statement

The datasets generated for this study are available on request to the corresponding author.

## Ethics Statement

The studies involving human participants were reviewed and approved by the local ethics committee of the University of Amsterdam. The patients/participants provided their written informed consent to participate in this study.

## Author Contributions

MaV and EG conceived and designed the experiments. GF-G performed the experiments. GF-G, JT, MaV, DS, and EG analyzed the data. JT, MeV, and CS contributed materials and analysis tools. GF-G and MaV wrote the article. All authors contributed to the article and approved the submitted version.

## Conflict of Interest

The authors declare that the research was conducted in the absence of any commercial or financial relationships that could be construed as a potential conflict of interest.

## Publisher’s Note

All claims expressed in this article are solely those of the authors and do not necessarily represent those of their affiliated organizations, or those of the publisher, the editors and the reviewers. Any product that may be evaluated in this article, or claim that may be made by its manufacturer, is not guaranteed or endorsed by the publisher.

## Footnotes

1 The initial sample was 64 participants. 6 typical readers and 3 dyslexics were excluded due to poor task performance (chance levels suggesting they did not engage in the task) or problems during EEG data recording.

2
www.neurobs.com

3 As the present analysis requires equal number of epochs across participants, we chose to include 30 epochs to avoid excluding additional participants having an insufficient number of artifact-free epochs.

4 A broad alpha range was chosen instead of the lower alpha (8–10 Hz) and upper alpha (10–13 Hz) to account for individual variability observed in alpha peak frequencies.
