# Corrigendum: Graph Analysis of EEG Functional Connectivity Networks During a Letter-Speech Sound Binding Task in Adult Dyslexics

**DOI:** 10.3389/fpsyg.2021.828043

**Published:** 2022-01-05

**Authors:** Gorka Fraga-González, Dirk J. A. Smit, Melle J. W. Van der Molen, Jurgen Tijms, Cornelis J. Stam, Eco J. C. de Geus, Maurits W. Van der Molen

**Affiliations:** ^1^Department of Psychology, University of Amsterdam, Amsterdam, Netherlands; ^2^Rudolf Berlin Center, Amsterdam, Netherlands; ^3^Department of Child and Adolescent Psychiatry and Psychotherapy, University Hospital of Psychiatry Zurich, University of Zurich, Zurich, Switzerland; ^4^Amsterdam Neuroscience, Amsterdam UMC, Amsterdam, Netherlands; ^5^Neuroscience Campus Amsterdam, VU University, Amsterdam, Netherlands; ^6^Institute of Psychology, Leiden University, Leiden, Netherlands; ^7^Leiden Institute for Brain and Cognition, Leiden University, Leiden, Netherlands; ^8^RID Institute, Amsterdam, Netherlands; ^9^Department of Clinical Neuropsychology and MEG Center, VU University Medical Center, Amsterdam, Netherlands; ^10^Amsterdam Brain and Cognition, University of Amsterdam, Amsterdam, Netherlands

**Keywords:** EEG, networks, dyslexia, letter-speech sound associations, phase lag index, minimum spanning tree (MST)

In the original article, we neglected to include the funder, University of Zurich, UZH Postdoc Grant, grant no. [FK-19-040] to GF-G. The corrected Funding statement appears below.

## Funding

This project was part of the research program “Characterization of functional brain network organization in dyslexia and development” funded by the Amsterdam Brain and Mind Project, a UvA-VUA Amsterdam Academic Alliance Initiative (https://www.abmp.eu/). Additional funding was received from University of Zurich, UZH Postdoc Grant, grant no. [FK-19-040] to GF-G.

In the original article the email address of the corresponding author was incorrect. The correct email address is gorka.fragagonzalez@uzh.ch.

The authors apologize for this error and state that this does not change the scientific conclusions of the article in any way. The original article has been updated.

## Publisher's Note

All claims expressed in this article are solely those of the authors and do not necessarily represent those of their affiliated organizations, or those of the publisher, the editors and the reviewers. Any product that may be evaluated in this article, or claim that may be made by its manufacturer, is not guaranteed or endorsed by the publisher.

