# Supplementary material for: Graph Analysis of EEG Functional Connectivity Networks During a Letter-Speech Sound Binding Task in Adult Dyslexics
Source: Front Psychol. 2021 Nov 19;12:767839. doi: 10.3389/fpsyg.2021.767839 (PMC8658451; doi:10.3389/fpsyg.2021.767839)

# Appendix

| Table. A.1. List of stimuli used in the task. Visual and auditory stimuli were randomly associated and pairs were randomly drawn for each block. | | |  |
| --- | --- | --- | --- |
|  | | |  |
| Symbols (Georgian) |  | Phonemes |  |
| Ⴜ |  | ui [œʏ̯] |  |
| Ⴁ |  | s [s] |  |
| Ⴂ |  | ei [ɛɪ̯] |  |
| Ⴃ |  | oei [uɪ̯] |  |
| Ⴟ |  | n [n] |  |
| Ⴊ |  | r [r] |  |
| Ⴡ |  | f [f] |  |
| Ⴇ |  | j [j] |  |
| Ⴌ |  | g [χ] |  |
| Ⴉ |  | l [l] |  |
| Ⴖ |  | w [ʋ] |  |
| Ⴏ |  | h [h] |  |
| Ⴐ |  | a [ɑ] |  |
| Ⴕ |  | k [k] |  |
| Ⴒ |  | u [ʏ] |  |
| Ⴔ |  | o [[ɔ](https://en.wikipedia.org/wiki/Open-mid_back_rounded_vowel)] |  |
|  |  |  |  |

| **Table A.2.** Task performance in letter-speech sound task for the consistent trials. | | | | | | | | | | |
| --- | --- | --- | --- | --- | --- | --- | --- | --- | --- | --- |
|  |  |  | Typical readers (N = 31) | | |  | Dyslexics (N = 24) | | |  |
|  |  |  | Accuracy |  | Reaction time |  | Accuracy |  | Reaction time |  |
|  |  |  | *M(SD)* |  | *M(SD)* |  | *M(SD)* |  | *M(SD)* |  |
|  |  |  |  |  |  |  |  |  |  |  |
| Letter-speech sound binding task | *Block 1* | Bin1 | 79.35 (14.65) |  | 915.86 (147.63) |  | 72.00 (16.97) |  | 985.39 (167.59) |  |
|  |  | Bin2 | 88.90 (9.94) |  | 894.48 (138.00) |  | 81.50 (18.42) |  | 954.40 (183.96) |  |
|  |  | Bin3 | 88.52 (13.85) |  | 916.74 (151.92) |  | 86.50 (13.49) |  | 967.18 (148.23) |  |
|  |  | Bin4 | 87.23 (11.29) |  | 904.54 (149.75) |  | 90.83 (11.47) |  | 952.24 (153.96) |  |
|  |  |  |  |  |  |  |  |  |  |  |
|  | *Block 2* | Bin1 | 74.06 (16.19) |  | 880.20 (164.01) |  | 72.67 (15.14) |  | 951.47 (159.93) |  |
|  |  | Bin2 | 85.81 (15.10) |  | 862.72 (175.21) |  | 87.50 (10.89) |  | 944.85 (150.31) |  |
|  |  | Bin3 | 86.45 (11.85) |  | 859.62 (209.68) |  | 91.00 (6.80) |  | 957.61 (149.98) |  |
|  |  | Bin4 | 87.87 (14.48) |  | 839.13 (185.59) |  | 89.33 (12.18) |  | 961.15 (135.07) |  |
|  |  |  |  |  |  |  |  |  |  |  |
|  | *Block 3* | Bin1 | 77.94 (14.38) |  | 853.01 (132.94) |  | 78.17 (14.10) |  | 906.51 (159.51) |  |
|  |  | Bin2 | 85.68 (14.41) |  | 822.07 (191.49) |  | 88.33 (13.80) |  | 916.42 (167.24) |  |
|  |  | Bin3 | 86.06 (16.16) |  | 818.25 (215.76) |  | 87.00 (12.77) |  | 896.29 (170.33) |  |
|  |  | Bin4 | 86.06 (13.66) |  | 842.11 (180.26) |  | 87.50 (10.37) |  | 903.78 (153.14) |  |
|  |  |  |  |  |  |  |  |  |  |  |
|  | *Block 4* | Bin1 | 75.87 (14.59) |  | 897.45 (142.09) |  | 76.00 (14.59) |  | 869.91 (159.16) |  |
|  |  | Bin2 | 86.45 (11.62) |  | 866.43 (145.63) |  | 86.17 (14.68) |  | 922.12 (190.80) |  |
|  |  | Bin3 | 89.68 (10.26) |  | 878.62 (141.50) |  | 85.83 (12.76) |  | 909.55 (159.83) |  |
|  |  | Bin4 | 88.39 (11.52) |  | 858.42 (127.41) |  | 88.67 (14.09) |  | 966.19 (166.46) |  |
|  |  |  |  |  |  |  |  |  |  |  |
|  | Mean | Bin1 | 76.81 (9.90) |  | 886.63 (124.73) |  | 74.71 (9.53) |  | 928.32 (131.80) |  |
|  |  | Bin2 | 86.71 (8.65) |  | 861.42 (138.27) |  | 85.88 (10.27) |  | 934.45 (134.80) |  |
|  |  | Bin3 | 87.68 (9.80) |  | 868.31 (144.24) |  | 87.58 (8.27) |  | 932.66 (118.45) |  |
|  |  | Bin4 | 87.39 (9.92) |  | 861.05 (131.42) |  | 89.08 (9.22) |  | 945.84 (112.73) |  |
|  |  |  |  |  |  |  |  |  |  |  |
| Bin 1 = trials 1 to 25; Bin2 = trials 26 to 50; Bin3 = trials 51 to 75; Bin4 = trials 76 to 100.  Reaction times to correct responses in milliseconds. Accuracy = percentage of correct responses. | | | | | | | | | | |

| **Table A.3.** Group and condition comparisons for PLI and MST metrics. | | | | | | | | | | | | | |
| --- | --- | --- | --- | --- | --- | --- | --- | --- | --- | --- | --- | --- | --- |
|  |  |  |  | Within-subjects | | | | |  | | Between-subjects |  | |
|  |  |  |  | *Condition* | | |  | *Condition x Dyslexia* |  | | *Dyslexia* |  | |
|  |  |  | ΔTask | *F* | *p* | *η^2^* |  | *F* | *p* |  | *F* | *p* |  |
|  |  |  |  |  |  |  |  |  |  |  |  |  |  |
| *Theta* | PLI |  |  | 2.12 | .151 | 0.04 |  | **4.45** | **.040** |  | 1.04 | .313 |  |
|  | Degree |  | ↓ | **18.06** | **.000^**^** | **0.26** |  | *3.45* | *.069* |  | 1.60 | .211 |  |
|  | Leaf |  | ↓ | **59.98** | **.000^**^** | **0.53** |  | *3.60* | *.064* |  | 1.10 | .163 |  |
|  | Diameter |  | ↑ | **23.63** | **.000^**^** | **0.31** |  | 1.51 | .224 |  | 0.00 | .962 |  |
|  | Ecc |  | ↑ | **25.90** | **.000^**^** | **0.32** |  | 2.25 | .140 |  | 0.00 | .977 |  |
|  | *BC* |  | ↓ | 1.50 | .225 | 0.03 |  | 0.52 | .476 |  | 0.33 | .569 |  |
|  | *T_H_* |  | ↓ | **49.70** | **.000^**^** | **0.48** |  | **2.09** | **.038** |  | 2.47 | .122 |  |
|  | *R* |  | ↓ | **42.86** | **.000^**^** | **0.44** |  | *3.20* | *.080* |  | **6.36** | **.015** |  |
|  | Kappa |  | ↓ | **26.82** | **.000^**^** | **0.34** |  | 2.13 | .125 |  | 1.22 | .275 |  |
|  | MST Mean |  | ↑ | **22.29** | **.000^**^** | **0.30** |  | **5.70** | **.021** |  | 0.90 | .766 |  |
|  |  |  |  |  |  |  |  |  |  |  |  |  |  |
| *Alpha* | PLI |  | ↓ | **29.03** | **.000^**^** | **0.35** |  | 2.25 | .140 |  | 2.50 | .119 |  |
|  | Degree |  | ↓ | **74.02** | **.000^**^** | **0.58** |  | **4.09** | **.048** |  | 1.29 | .261 |  |
|  | Leaf |  | ↓ | **111.26** | **.000^**^** | **0.68** |  | 0.90 | .364 |  | 0.39 | .537 |  |
|  | Diameter |  | ↑ | **38.77** | **.000^**^** | **0.42** |  | 1.10 | .298 |  | 1.65 | .205 |  |
|  | Ecc |  | ↑ | **38.15** | **.000^**^** | **0.42** |  | 1.27 | .265 |  | 1.55 | .218 |  |
|  | *BC* |  | ↓ | **12.54** | **.000^**^** | **0.19** |  | 2.20 | .144 |  | 0.96 | .333 |  |
|  | *T_H_* |  | ↓ | **51.76** | **.000^**^** | **0.49** |  | 0.05 | .825 |  | 0.04 | .842 |  |
|  | *R* |  | ↓ | **67.63** | **.000^**^** | **0.56** |  | 0.87 | .355 |  | 0.06 | .800 |  |
|  | Kappa |  | ↓ | **98.80** | **.000^**^** | **0.65** |  | **5.68** | **.021** |  | 1.47 | .230 |  |
|  | MST Mean ^a^ |  | ↓ | **15.21** | **.000^**^** | **0.22** |  | 2.63 | .111 |  | *3.30* | *.075* |  |
|  |  |  |  |  |  |  |  |  |  |  |  |  |  |
| *Beta* | PLI |  | ↓ | **24.64** | **.000^**^** | **0.32** |  | 2.66 | .109 |  | *3.10* | *.084* |  |
|  | Degree |  | ↓ | **26.47** | **.000^**^** | **0.33** |  | 0.25 | .620 |  | 0.16 | .694 |  |
|  | Leaf |  | ↓ | **49.01** | **.000^**^** | **0.48** |  | 0.00 | .934 |  | 0.53 | .472 |  |
|  | Diameter |  | ↑ | **18.17** | **.000^**^** | **0.26** |  | 0.09 | .772 |  | 0.08 | .779 |  |
|  | Ecc |  | ↑ | **17.20** | **.000^**^** | **0.25** |  | 0.03 | .853 |  | 0.15 | .704 |  |
|  | *BC* |  | ↓ | **10.48** | **.002^**^** | **0.16** |  | 0.28 | .600 |  | 0.04 | .845 |  |
|  | *T_H_* |  | ↓ | **23.16** | **.000^**^** | **0.30** |  | 0.04 | .842 |  | 0.40 | .842 |  |
|  | *R* |  | ↓ | **38.20** | **.000^**^** | **0.42** |  | 0.01 | .911 |  | 0.29 | .590 |  |
|  | Kappa |  | ↓ | **30.88** | **.000^*^** | **0.37** |  | 0.11 | .743 |  | 0.41 | .523 |  |
|  | MST Mean |  | . | *3.42* | *.070* | *0.06* |  | **5.27** | **.026** |  | 1.04 | .312 |  |
|  |  |  |  |  | |  | |  |  | |  |  | |
| *Note.* PLI, phase lag index; Ecc*,* Eccentricity; *BC*, betweenness centrality; *T_H_*, tree hierarchy; R, degree correlation; ↑ indicates increase in task *vs* baselines; ↓ indicates decrease in task *vs* baselines; ^a^ direction of effect differs between frequency bands;  ***** significant effects after FDR correction at *q* = 0.10; ****** significant effects after FDR correction at *q* = 0.05; italic text represents trends; bold text represents significant effects at uncorrected p < 0.05. | | | | | | | | | | | | | |

| **Table A.4.** Group comparisons in task and baseline for network metrics in the theta and alpha bands. | | | | | | | | | | |
| --- | --- | --- | --- | --- | --- | --- | --- | --- | --- | --- |
|  |  |  | *Task* | | |  | *Baseline* | | |  |
|  |  |  | *F* | *p* | *Dys vs Typ* |  | *F* | *p* | *Dys vs Typ* |  |
|  |  |  |  |  |  |  |  |  |  |  |
| *Theta* | Degree |  | *3.92* | *.053* |  |  | 0.05 | .819 |  |  |
|  | Leaf |  | *3.63* | *.062* |  |  | 0.19 | .668 |  |  |
|  | Diameter |  | 0.52 | .473 |  |  | 0.47 | .496 |  |  |
|  | Ecc |  | 0.67 | .416 |  |  | 0.70 | .407 |  |  |
|  | *BC* |  | 0.86 | .358 |  |  | 0.02 | .890 |  |  |
|  | *T_H_* |  | *3.91* | *.053* |  |  | 0.37 | .544 |  |  |
|  | *R* |  | **7.33** | **.009*** | < |  | 1.23 | .272 |  |  |
|  | Kappa |  | 2.83 | .099 |  |  | 0.00 | .962 |  |  |
|  | MST Mean |  | 1.48 | .230 |  |  | 1.88 | .176 |  |  |
|  |  |  |  |  |  |  |  |  |  |  |
| *Alpha* | Degree |  | 0.77 | .383 |  |  | *3.10* | *.084* |  |  |
|  | Leaf |  | 0.02 | .880 |  |  | 1.11 | .297 |  |  |
|  | Diameter |  | 0.24 | .629 |  |  | 2.62 | .112 |  |  |
|  | Ecc |  | 0.17 | .681 |  |  | 2.70 | .106 |  |  |
|  | *BC* |  | 0.01 | .917 |  |  | 2.45 | .123 |  |  |
|  | *T_H_* |  | 0.07 | .792 |  |  | 0.00 | .959 |  |  |
|  | *R* |  | 0.06 | .808 |  |  | 0.59 | .446 |  |  |
|  | Kappa |  | 0.00 | .948 |  |  | *3.71* | *.060* |  |  |
|  | *MST Mean* |  | **5.88** | **.019** | < |  | *3.87* | *.054* |  |  |
|  | | | | | | | | | | |
| *Note.* PLI, phase lag index; *Dys*, dyslexics; *Typ,* typical readers; Ecc*,* Eccentricity; *BC*, betweenness centrality; *T_H_*, tree hierarchy; R, degree correlation. * = significant effects after FDR correction at *q* = 0.10; italic text represents trends; bold text represents significant effects at uncorrected *p* < 0.05. | | | | | | | | | | |

| **Table A.5.** Significant stepwise regressions of performance, age and cognitive skills to EEG metrics in both groups. | | | | | | | | |
| --- | --- | --- | --- | --- | --- | --- | --- | --- |
|  |  |  | SE | Adj. *R^2^* | *ΔR^2^* |  | *F change* |  |
|  |  |  |  |  |  |  |  |  |
| Mean RT | *Model 1* | Task Beta Diameter | 119.85 | .109 | .125 |  | 7.57^*^ |  |
|  | *Model 2* | + Task theta power | 113.99 | .193 | .098 |  | 6.58^*^ |  |
|  |  |  |  |  |  |  |  |  |
| Mean Accuracy | *Model 1* | Baseline Beta BC | 8.26 | .094 | .111 |  | 6.62^**^ |  |
|  | *Model 2* | + Baseline Alpha Ecc | 7.88 | .176 | .095 |  | 6.25^*^ |  |
|  |  |  |  |  |  |  |  |  |
| Age | *Model 1* | Task Alpha R | 2.29 | .111 | .128 |  | 7.75^**^ |  |
|  | *Model 2* | + Baseline Theta Th | 2.21 | .170 | .073 |  | 4.77^*^ |  |
|  |  |  |  |  |  |  |  |  |
| RAN total | *Model 1* | Baseline Alpha Kappa | 4.08 | .087 | .104 |  | 6.13^*^ |  |
|  | *Model 2* | + Task Beta MST Mean | 3.88 | .177 | .104 |  | 6.79^*^ |  |
|  |  |  |  |  |  |  |  |  |
| RAN letters | *Model 1* | Baseline Alpha Degree | 4.33 | .088 | .104 |  | 6.18^*^ |  |
|  | *Model 2* | + Task Alpha FFT power | 4.15 | .164 | .091 |  | 5.86^*^ |  |
|  |  |  |  |  |  |  |  |  |
| RAN numbers | *Model 1* | Baseline Alpha Degree | 4.05 | .088 | .105 |  | 6.21^*^ |  |
|  | *Model 2* | +Task Theta Th | 3.88 | .165 | .091 |  | 5.91^*^ |  |
|  | *Model 3* | + Task Theta Ecc | 3.65 | .258 | .103 |  | 7.47^**^ |  |
|  | *Model 4* | + Baseline Beta BC | 3.54 | .302 | .055 |  | 4.26^*^ |  |
|  |  |  |  |  |  |  |  |  |
| RAN colors | *Model 1* | Baseline Beta BC | 5.11 | .061 | .079 |  | 4.52^*^ |  |
|  |  |  |  |  |  |  |  |  |
| RAN images | *Model 1* | Task Alpha Th | 6.14 | .149 | .165 |  | 10.45^**^ |  |
|  | *Model 2* | + Task Theta Th | 5.66 | .277 | .139 |  | 10.36^**^ |  |
|  | *Model 3* | + Task Beta PLI | 5.47 | .323 | .057 |  | 4.55^*^ |  |
|  |  | | | | | | | |
| SE = standard error of the estimate; Adj. *R^2^ =* adjusted R squared; *ΔR^2^=* change in R squared; + indicates variable is added to those of preceding models. BC = betweenness centrality; PLI = phase lag index; Th = tree hierarchy; R = degree correlation; Ecc = eccentricity. ^*^ *p* < 0.05; ^**^ *p* < 0.01; ^***^ *p* < 0.001. | | | | | | | | |


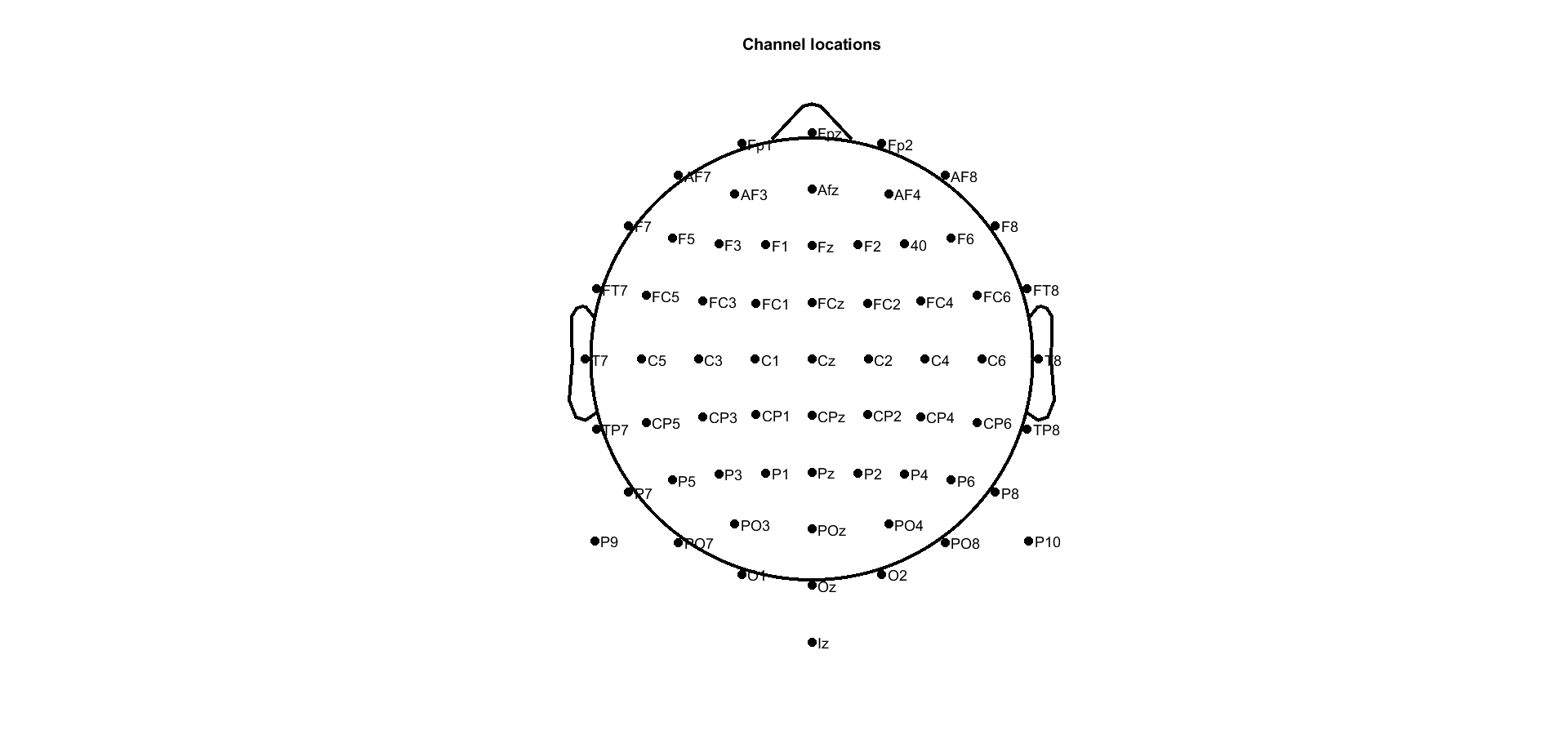


Figure A.1. Location of the 64 scalp electrodes

Figure A.2. Task performance in the consistent trials. Left panel: percentage of correct responses averaged across blocks per bins of 25 trials for typical (blue lines) and dyslexic readers (red lines). Right panel: RT averaged across blocks for typical (blue bars) and dyslexic readers (red bars). Asterisk represents significant differences between pairs of bins at *p* < 0.05.


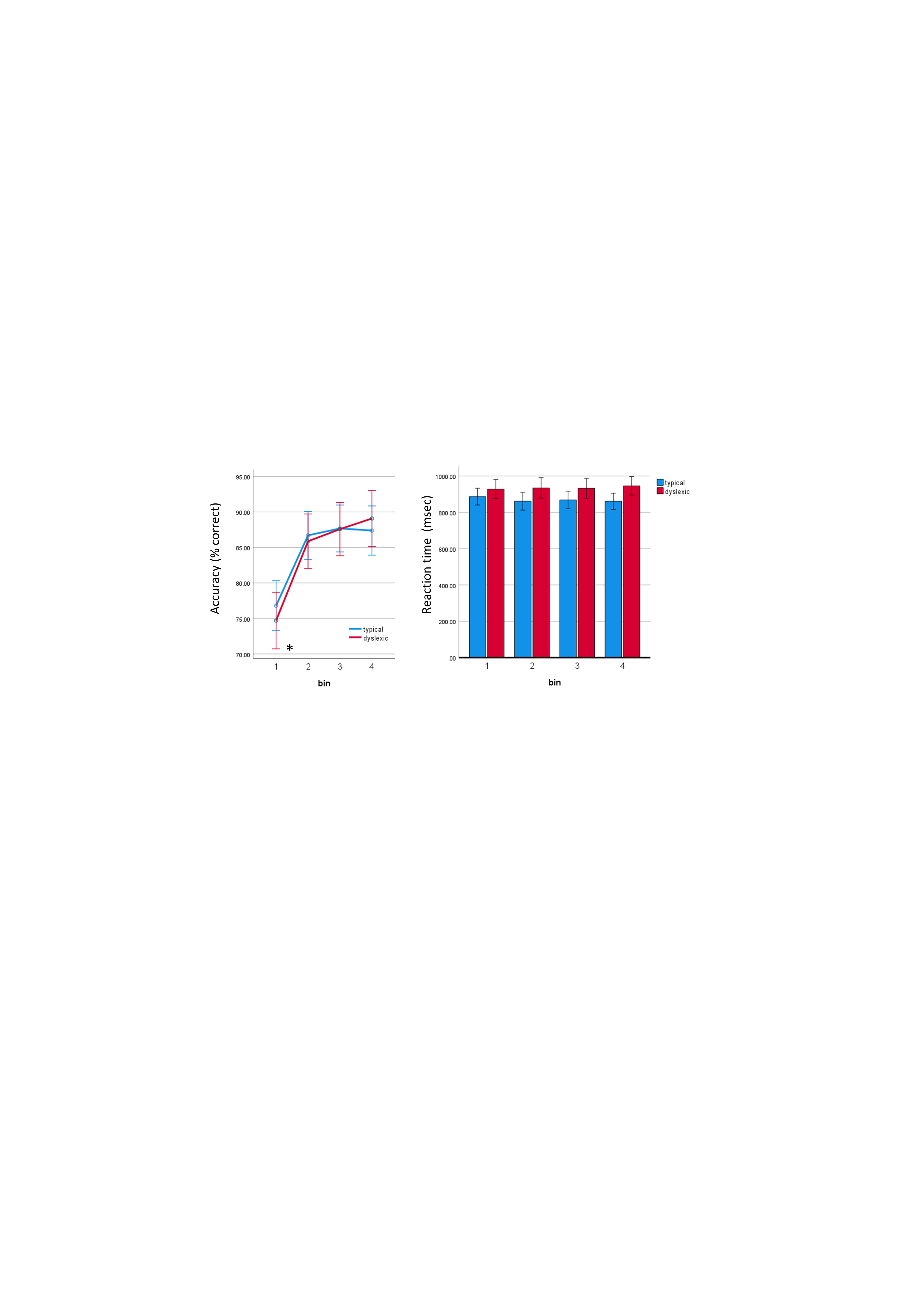


Figure A.3. Averaged PLI in the theta band (A) Mean PLI plotted against mean relative power. Dashed lines are regression lines for typical readers (blue) and dyslexics (red). (B) Mean PLI for task and baseline recordings. DYS = dyslexics, TYP = typical readers. Error bars indicate 95% CI.


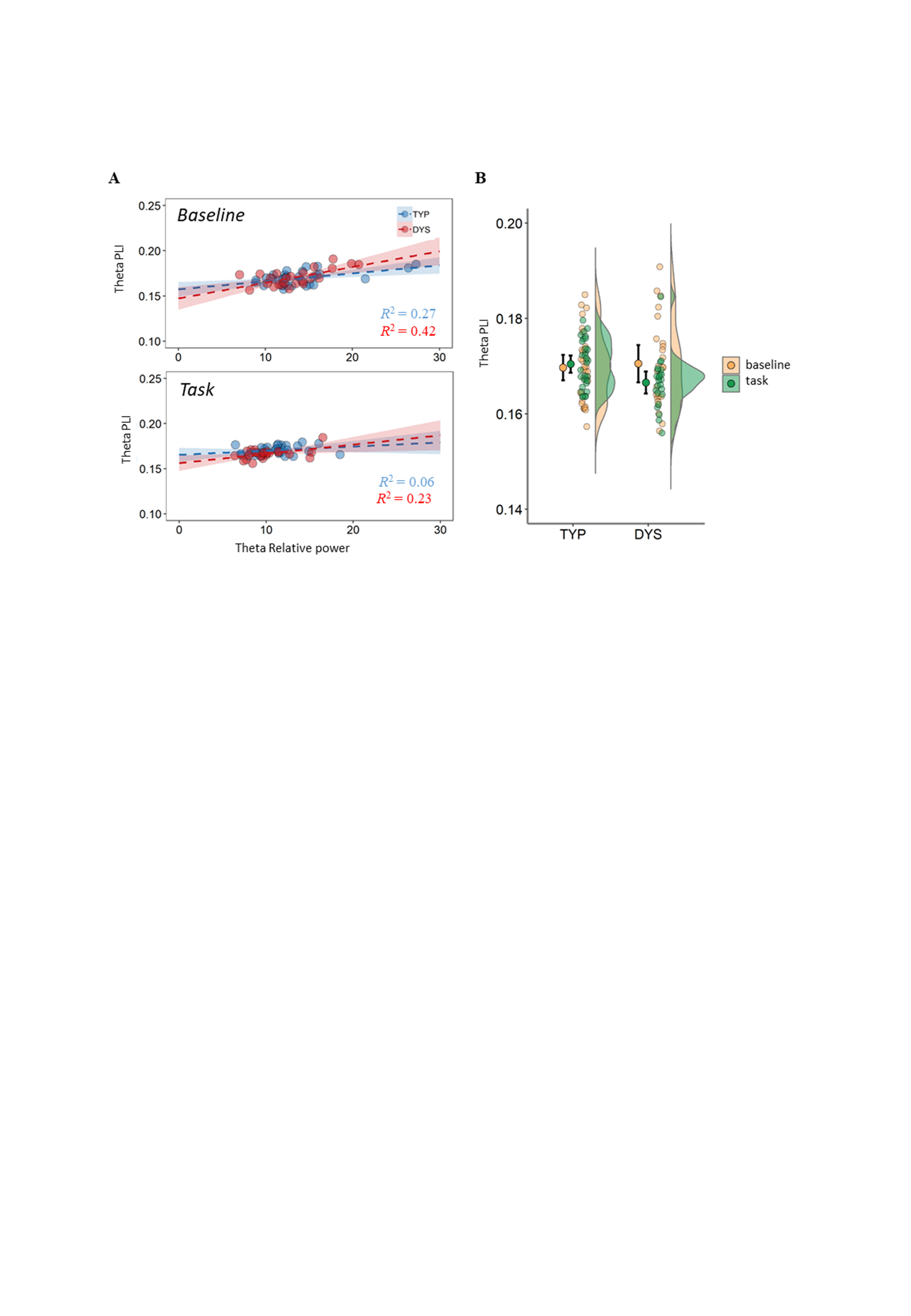

Supplement: Supplementary file 1 [file Data_Sheet_1.docx]
